# Competitive adsorption of microRNA-532-3p by circular RNA SOD2 activates Thioredoxin Interacting Protein/NLR family pyrin domain containing 3 pathway and promotes pyroptosis of non-alcoholic fatty hepatocytes

**DOI:** 10.1186/s40001-024-01817-4

**Published:** 2024-04-24

**Authors:** FengJuan Chen, YuFeng Xing, ZhiJie Chen, XiaoMan Chen, Jie Li, Si Gong, Fang Luo, QingXian Cai

**Affiliations:** 1 Department of Hepatopathy, The Third People’s Hospital of Shenzhen, The Second Affiliated Hospital of Southern University of Science and Technology, No. 29, Bulan Road, Longgang District, Shenzhen City, 518112 Guangdong Province China; 2 Department of Hepatopathy, Shenzhen Traditional Chinese Medicine Hospital, Shenzhen City, 518033 Guangdong Province China; 3 Department of Infectious Diseases, Third Affiliated Hospital of Sun Yat-sen University, Guangzhou City, 510630 Guangdong Province China

**Keywords:** Circular RNA SOD2, MicroRNA-532-3p, Non-alcoholic fatty liver disease, Pyroptosis

## Abstract

**Objective:**

There is a growing body of evidence indicating that pyroptosis, a programmed cell death mechanism, plays a crucial role in the exacerbation of inflammation and fibrosis in the pathogenesis of non-alcoholic fatty liver disease (NAFLD). Circular RNAs (circRNAs), functioning as vital regulators within NAFLD, have been shown to mediate the process of cell pyroptosis. This study aims to elucidate the roles and mechanisms of circRNAs in NAFLD.

**Methods:**

Utilizing a high-fat diet (HFD)-induced rat model for in vivo experimentation and hepatocytes treated with palmitic acid (PA) for in vitro models, we identified circular RNA SOD2 (circSOD2) as our circRNA of interest through analysis with the circMine database. The expression levels of associated genes and pyroptosis-related proteins were determined using quantitative real-time polymerase chain reaction and Western blotting, alongside immunohistochemistry. Serum liver function markers, cellular inflammatory cytokines, malondialdehyde, lactate dehydrogenase levels, and mitochondrial membrane potential, were assessed using enzyme-linked immunosorbent assay, standard assay kits, or JC-1 staining. Flow cytometry was employed to detect pyroptotic cells, and lipid deposition in liver tissues was observed via Oil Red O staining. The interactions between miR-532-3p/circSOD2 and miR-532-3p/Thioredoxin Interacting Protein (TXNIP) were validated through dual-luciferase reporter assays and RNA immunoprecipitation experiments.

**Results:**

Our findings demonstrate that, in both in vivo and in vitro NAFLD models, there was an upregulation of circSOD2 and TXNIP, alongside a downregulation of miR-532-3p. Mechanistically, miR-532-3p directly bound to the 3'-UTR of TXNIP, thereby mediating inflammation and cell pyroptosis through targeting the TXNIP/NLR family pyrin domain containing 3 (NLRP3) inflammasome signaling pathway. circSOD2 directly interacted with miR-532-3p, relieving the suppression on the TXNIP/NLRP3 signaling pathway. Functionally, the knockdown of circSOD2 or TXNIP improved hepatocyte pyroptosis; the deletion of miR-532-3p reversed the effects of circSOD2 knockdown, and the deletion of TXNIP reversed the effects of circSOD2 overexpression. Furthermore, the knockdown of circSOD2 significantly mitigated the progression of NAFLD in vivo.

**Conclusion:**

circSOD2 competitively sponges miR-532-3p to activate the TXNIP/NLRP3 inflammasome signaling pathway, promoting pyroptosis in NAFLD.

**Supplementary Information:**

The online version contains supplementary material available at 10.1186/s40001-024-01817-4.

## Introduction

Non-alcoholic fatty liver disease (NAFLD), a major contributor to chronic liver disorders, stands as one of the globally pervasive diseases [1, 2]. Characterized by excessive fat deposition in the liver not associated with alcohol intake [3], NAFLD can progress from its earliest stage of hepatic steatosis, typically self-limiting, to non-Alcoholic Steatohepatitis (NASH), and subsequently to cirrhosis and hepatocellular carcinoma (HCC) [4]. In clinical settings, management strategies for NAFLD include lifestyle modifications and pharmacological interventions [5]. However, adherence to these interventions is significantly challenged by poor patient compliance and the adverse effects associated with current pharmacological options [6].

Research identifies insulin resistance, dysfunction of adipose tissue, stress in mitochondria and endoplasmic reticulum, chronic inflammation, and both genetic and epigenetic factors as key determinants in the susceptibility and advancement of NAFLD [7]. Hormonal imbalances, including those in insulin, sex hormones (notably, changes in estrogen and testosterone levels), thyroid hormone levels, and adrenal cortex hormones, are implicated in the dysregulation of lipid metabolism and the accumulation of fat within the liver. Such imbalances not only promote hepatic fat accumulation but also alter the synthesis and breakdown of fatty acids, exacerbating the disease trajectory of NAFLD [8]. Pyroptosis, a form of programmed cell death characterized by cellular swelling until rupture, triggering a potent inflammatory response, has been recognized as a critical factor in the evolution of NAFLD. This process can lead to the activation of hepatic stellate cells and fibrogenesis, further fostering inflammatory responses within the liver [9]. Despite these insights, the interactions among these factors, their detailed mechanisms, and the relationships between upstream regulators and downstream effects remain inadequately defined. Thus, it is imperative to elucidate the underlying mechanisms of NAFLD to facilitate the development of novel therapeutic approaches.

Circular RNAs (circRNAs), distinguishable from conventional linear RNAs by their covalently closed loop structures, were once considered non-functional splicing by-products [10]. Recent evidence, however, underscores their crucial role in gene transcription through complex formation with RNA binding proteins or microRNAs (miRNAs), linking circRNAs to an array of human diseases. Emerging data increasingly indicate the involvement of circRNAs in the pathogenesis of various liver conditions, including NAFLD, NASH, cirrhosis, and HCC [11, 12]. For instance, circ_0057558 has been shown to facilitate the development of NAFLD by targeting miR-206 and modulating the Rho-associated coiled-coil containing protein kinase 1/AMP-activated protein kinase (AMPK) signaling pathway [6]. Similarly, the circRNA_002581-miR-122-cytoplasmic polyadenylation element-binding 1 axis is actively involved in the pathogenesis of NASH through its engagement with the Phosphatase and tensin homolog-AMPK-mTOR pathway and related autophagy suppression [13]. Despite these advancements, the functional landscape of circRNAs in NAFLD remains a burgeoning field of inquiry necessitating further investigation. In this study, our analysis via the circMine database revealed both upregulation (107 circRNAs) and downregulation (181 circRNAs) in the expression of circRNAs in tissue samples from patients with NASH cirrhosis compared to normal liver tissue samples, with circSOD2 exhibiting significant upregulation. Although prior research has implicated circSOD2 in the development and progression of cancer [14, 15], its role and mechanisms in NAFLD progression have not been reported.

The concept of the “sponge effect” of circRNAs necessitates the identification of their downstream miRNA–mRNA targets for functional elucidation [16]. In our study, predictive analysis of miRNA target sites revealed a presumptive binding site for miR-532-3p within the 3ʹ untranslated region (UTR) of circSOD2, indicating a direct interaction. Additionally, a binding site for miR-532-3p was observed in the 3ʹ UTR of thioredoxin-interacting protein (TXNIP) mRNA. Previous research has established the relevance of TXNIP levels to NAFLD [17] and its contribution to the progression of NASH [18]. We hypothesize that circSOD2, acting as an miRNA sponge, could regulate TXNIP expression by binding with miR-532-3p, thereby exacerbating the progression of NAFLD. Employing a high-fat diet (HFD)-fed mouse model for in vivo experimentation and palmitic acid (PA)-treated hepatocytes for in vitro analysis, our study extensively explored the pivotal role of the circSOD2/miR-532-3p/TXNIP/NLRP3 axis in NAFLD, offering novel molecular targets for therapeutic strategy development against this condition.

## Materials and methods

### Clinical sample acquisition

From 2014 to 2017, liver biopsy specimens were procured from 58 adult patients diagnosed with NAFLD presenting with simple steatosis, non-severe fibrosis, or NASH-related cirrhosis at Third People’s Hospital of Shenzhen. Exclusions applied to hepatic conditions stemming from viral, autoimmune, cholestasis, genetic, alcoholic, and drug-induced etiologies. Concomitantly, normal liver tissues were harvested from 43 individuals undergoing hepatic hemangioma resection at Third People’s Hospital of Shenzhen. The cohort comprised an average age of 47, with a gender distribution of 36 males and 22 females. Liver specimen evaluation was independently conducted by two hepatopathologists blind to patient details, adhering to the American Association for the Study of Liver Diseases’ 2018 Practice Guidelines. Ethical compliance was secured through written informed consent from all subjects, institutional review board approval from Third People's Hospital of Shenzhen (Approval number: 2022-044), and adherence to the Declaration of Helsinki. Patient clinical information is shown in (Additional file 3: Table S2).

### Cell culture procedures

The murine hepatocyte cell line, AML12, acquired from the China Center for Type Culture Collection (Shanghai, China), was cultured in Dulbecco’s Modified Eagle Medium/Nutrient Mixture F-12 (DMEM-F12) enriched with 10% Fetal Bovine Serum (BI), 1% Insulin–Transferrin–Selenium (ITS, Sciencell, USA), 0.2% Dexamethasone (Sigma-Aldrich, USA), and 1% Penicillin–Streptomycin solution (Sigma-Aldrich). These cells were maintained under a humidified atmosphere of 5% CO_2_ at 37 °C.

### Actinomycin D and RNase R treatments

To ascertain the stability of circSOD2 within AML12 cells, we employed treatments with Actinomycin D and RNase R. AML12 cells (1 × 10^6^) were incubated in complete medium supplemented with 2 μg/mL Actinomycin D (Sigma) or Dimethyl Sulfoxide (Sigma) as a control. For RNA digestion, total RNA from AML12 cells (1 × 10^5^) was incubated with 3 Units/μg RNase R (Geneseed, Guangzhou, China) or Diethyl Pyrocarbonate-treated water (Sigma) as a control. circSOD2 and linear Glyceraldehyde 3-Phosphate Dehydrogenase (GAPDH) mRNA were quantified via quantitative Reverse Transcription Polymerase Chain Reaction (RT-qPCR).

### Northern blot analysis

Total RNA from AML12 cells, including RNase R-treated samples, were subjected to Northern blot analysis utilizing the NorthernMax^®^ Kit (Invitrogen, Life Technologies Inc., Germany). Briefly, samples were electrophoresed on a 1% Formaldehyde–Polyacrylamide–Urea gel, transferred to a positively charged Hybond N^+^ membrane (Amersham), and cross-linked via ultraviolet irradiation. Hybridization ensued overnight at 50 °C with 3ʹ-Digoxigenin-labeled probes targeting circSOD2, procured from Axl-Bio (Guangzhou, China).

### Subcellular fractionation

The PARIS™ Kit (Thermo Fisher Scientific) facilitated the assessment of circSOD2's subcellular localization. AML12 cells were lysed, and subsequent centrifugation at 12,000×*g* yielded supernatant and nuclear pellets for cytoplasmic and nuclear RNA extraction, respectively. RT-qPCR evaluated circSOD2 presence within cytoplasmic and nuclear fractions, utilizing 18S rRNA and U6 small nuclear RNA as respective compartmental controls.

### Fluorescence in situ hybridization (FISH)

Fluorescein isothiocyanate-labeled circSOD2 and Cy3-labeled miR-532-3p probes, supplied by Ribobio (Guangzhou, China), facilitated co-localization analysis within cells. Post-seeding AML12 cells (1 × 10^5^ cells/well) in 6-well plates and achieving 80% confluency, cells were fixed with 4% paraformaldehyde. Pre-hybridization and hybridization processes were conducted at 42 °C, initially in pre-hybridization solution for 1 h, followed by overnight incubation with probes (300 ng/mL) in hybridization solution. 4'-6-diamidino-2-phenylindole (DAPI) diluted in PBST (1:800) served for nuclear staining, with anti-fade mounting medium application preceding fluorescence microscopy examination.

### CircRNA immunoprecipitation (circRIP)

Biotinylated circSOD2 and control probes, synthesized by Invitrogen, underpinned circRIP experiments as previously delineated [19]. Following cell seeding in 10 cm dishes and a 48 h culture period, cells were fixed with 1% Formaldehyde, lysed, and ultrasonically disrupted. Post-centrifugation, 50 μL of supernatant was incubated with specific probe-linked streptavidin dynabeads at 37 °C for 3 h. Probe-dynabeads-circRNA complexes underwent washing and incubation with Lysis Buffer and Proteinase K to reverse Formaldehyde cross-linking. TRIzol reagent (Invitrogen) facilitated RNA extraction from the complexes, with RT-qPCR employed for purified RNA analysis.

### NASH cell model establishment

To emulate NASH in vitro, cells were cultured for 24 h in a 1 mM PA culture medium, constituted of 6 mL of 2.13 mg/mL PA, 5 mL of 220 mg/mL bovine serum albumin, and 39 mL of DMEM/F12 [20].

### Cell transfection

To modulate the expression of circSOD2 and TXNIP, small interfering RNAs (siRNAs) targeting circSOD2 (si-circSOD2#1, si-circSOD2#2) and TXNIP (si-TXNIP#1, si-TXNIP#2, si-NC) alongside overexpression plasmids (pcDNA3.1, pcDNA3.1-circSOD2) were constructed. For the regulation of miR-532-3p, both mimic and inhibitor constructs, alongside negative controls (mimic/inhibitor NC), were synthesized. These constructs and oligonucleotides were procured from RiboBio. Using Lipofectamine™ 2000 reagent (Invitrogen), AML12 cells were transiently transfected following the protocol of manufacturer, with transfection concentrations set at 40 nM for siRNAs, 40 nM for mimics, 20 nM for inhibitors, and 2 μg for plasmids. Transfection efficiency was evaluated after 48 h through RT-qPCR or Western blot. The sequences of siRNAs and the map of the pcDNA3.1 vector are provided in Additional file 2. Transfection efficiency, verified via Green Fluorescent Protein-tagged pcDNA3.1 vector and Cy3-labeled siRNAs, exceeded 80% as confirmed by flow cytometry (Additional file 1: Fig. S1).

### Flow cytometry

Pyroptosis in AML12 cells was quantified using the FAM-YVAD-FMK/Propidium Iodide (PI) assay kit (Cat#97, ImmunoChemistry, USA). Pyroptosis was characterized by cells positive for both cleaved caspase-1 (labeled with FAM-YVAD-FMK) and membrane pore formation (labeled with PI). A total of 1 × 10^5^ cells from suspension were incubated with FAM-YVAD-FMK at 37 °C in the dark for 1 h. Following centrifugation, cells were washed with washing buffer, incubated with 5μL PI for 15 min, and analyzed for pyroptotic cells using the BD FACSAria flow cytometer (Becton Dickinson and Company, USA).

### Lactate dehydrogenase (LDH) release assay

LDH activity released into the cell culture supernatant was determined using the CytoTox 96 Non-Radioactive Cytotoxicity Assay Kit (Promega, USA) according to the instructions of manufacturer. Briefly, 100 μL of cell culture medium was added to a 96-well plate. Test compounds and vehicle controls were added, and after incubation for the desired duration, lysis solution was optionally used to determine maximum LDH release control. Subsequently, 50 μL of the sample was transferred to a new 96-well plate, followed by the addition of 50 μL of CytoTox 96^®^ reagent to each well. After a 30-min incubation in the dark, 50 μL of stop solution was added to each well. The absorbance at 490 nm was recorded using a microplate reader.

### Mitochondrial membrane potential (MMP) assay

MMP was evaluated using the JC-1 dye assay kit (C2006, Beyotime Biotechnology) following the instructions of manufacturer. Post-transfection, cells were washed twice with PBS and incubated with JC-1 at 37 °C in the dark for 20 min. Unbound JC-1 dye was removed using PBS. The fluorescence of JC-1 monomers (green fluorescence) and aggregates (red fluorescence) was detected using a fluorescence microscope (Axio Observer, Zeiss), with monomeric forms measured at approximately 525 nm and aggregated forms at approximately 590 nm.

### Dual-luciferase reporter assay

To elucidate the interaction between circSOD2 and miR-532-3p, sequences containing miR-532-3p target sites from circSOD2 were cloned into the pMIR-REPORT™ luciferase reporter vector (Thermo Fisher Scientific Inc., Waltham, USA), creating wild-type circSOD2 (WT-circSOD2) constructs. Mutant variants of circSOD2 (MUT-circSOD2), lacking miR-532-3p complementary sites, were also generated. Co-transfection of these reporter constructs with mimic NC and miR-532-3p mimic into AML12 cells was followed by measurement of luciferase activity using the Dual-Luciferase Reporter Assay System (Promega, Madison, USA). Similarly, the complementarity between miR-532-3p and the 3'-UTR of TXNIP was assessed [21].

### RNA immunoprecipitation (RIP)

The interaction between miR-532-3p and TXNIP protein was determined using an RIP kit (Millipore, Billerica, MA) [22]. Lysed cells were centrifuged to collect the supernatant. A portion of the cell extract was reserved as input, and the remainder was incubated with antibodies for co-precipitation. Both the sample and Input were treated with proteinase K, and RNA was extracted for subsequent RT-qPCR detection of TXNIP.

### NAFLD model

Fifty male Sprague–Dawley rats (6 weeks old, 200 ± 20 g) were procured from Hunan Slack Experimental Animal Co., Ltd. (Hunan, China). These rats were housed under conditions of 24 ± 2 °C temperature, 50–60% humidity, and a 12 h light/dark cycle, with ad libitum access to food and water. After a 1-week acclimatization period, 15 rats were fed a standard diet (10% fat) as the control group, while 35 rats were fed a HFD (45% fat) to establish the NAFLD model. The standard diet and HFD were sourced from Ready Dietech (Shenzhen, China). After 12 weeks, 5 rats from each diet group were sacrificed, and hepatic steatosis was assessed via Oil Red O staining. The HFD rats were then randomly divided into three groups (10 rats each): NAFLD, sh-NC, and sh-circSOD2. The sh-NC and sh-circSOD2 groups received intravenous injections of the corresponding plasmids (0.2 mg/kg, once/week), while the NAFLD group received saline. After 4 weeks, rats were euthanized with pentobarbital sodium (30 mg/kg), blood was collected from the abdominal aorta and centrifuged to obtain serum, which was stored at –80 °C. The left lobe of the liver was snap-frozen in liquid nitrogen and stored at –80 °C, while part of the right lobe was fixed in 4% paraformaldehyde. Rat weights were measured weekly. This study received approval from the Third People’s Hospital of Shenzhen (No. 2022-SZ04) Animal Ethics Committee and was conducted in strict accordance with animal experimentation guidelines.

### Oil Red O staining

Oil Red O working solution was prepared for staining. Sections were rinsed with reverse osmosis water for 3 min, followed by a 30-s wash in 60% isopropanol, and stained with Oil Red O working solution for 20 min in the dark. Sections were then differentiated in 60% isopropanol for 30 s until the interstitium appeared transparent, rinsed with water for 1 min to remove residual isopropanol, and counterstained with Mayer's hematoxylin for 3 min. After a final RO water rinse, sections were mounted with glycerol gelatin and observed under a microscope.

### Terminal seoxynucleotidyl transferase dUTP nick end labeling (TUNEL) staining

Apoptosis in liver tissue was detected using TUNEL staining (Sevier, Wuhan, China). Paraffin sections were deparaffinized, rehydrated, treated with proteinase K for 20 min, and then incubated with a mixture of fluorescently labeled dUTP and TdT enzyme at 37 °C in a humidified chamber for 1 h. For positive controls, sections were treated with DNase I at room temperature (25 °C) for 10 min prior to the fluorescence labeling process. Negative controls were incubated with dUTP at room temperature (25 °C) for 10 min. Sections were developed with diaminobenzidine (DAB) and counterstained with DAPI. Dehydration with graded ethanol, clarification with xylene, and mounting with neutral balsam followed. Apoptotic cells were observed under a microscope (Olympus, Tokyo, Japan). The apoptosis rate was calculated as the number of apoptotic cells/total cell count × 100%.

### Biochemical marker detection

All biochemical markers were assayed strictly following the kit instructions. Tumor Necrosis Factor-alpha (TNF-α), Interleukin-1 beta (IL-1β), Interleukin-6 (IL-6), and Superoxide Dismutase (SOD) levels in cells and liver tissues were assessed using enzyme-linked immunosorbent assay (ELISA) kits (Beckman Coulter Life Sciences). Alanine aminotransferase (ALT), Aspartate aminotransferase (AST), total Cholesterol (TC), triglycerides (TG), and malondialdehyde (MDA) in rat serum, cells, and liver tissues were measured using standard biochemical assay kits (Nanjing Jiancheng Bioengineering Institute, China).

### Western blot

Total proteins were extracted from tissues and cells using pre-chilled radioimmunoprecipitation Assay lysis buffer at 4 °C. Protein concentrations were determined using the bicinchoninic acid assay kit (Pierce, Rockford, IL, USA). Following electrophoresis on a 12% sodium dodecyl sulphate polyacrylamide gel electrophoresis, 30 μg of protein samples were transferred to polyvinylidene difluoride membranes and blocked with 5% skim milk. The membranes were incubated overnight at 4 °C with primary antibodies against: GAPDH (ab8245, Abcam), TXNIP (14715, Cell Signaling Technology), NLRP3 (ab214185, Abcam), IL-1β (16806-1-AP, Proteintech), IL-18 (10663-1-AP, Proteintech), phosphorylated p65 (p-p65) (3033, Cell Signaling Technology), p65 (8242, Cell Signaling Technology), cleaved caspase-1 (ab179515, Abcam), and Gasdermin D (GSDMD) (ab209845, Abcam). Horseradish Peroxidase-conjugated secondary antibodies (1:1500; Jackson ImmunoResearch Laboratories, Inc., USA) were applied at room temperature for 1 h. Chemiluminescence was visualized using the enhanced chemiluminescence detection system and densitometric analysis was performed using Image Lab Software 5.1 (Bio-Rad Laboratories, USA).

### Immunohistochemistry (IHC)

IHC staining was conducted as previously described [23]. Deparaffinized liver tissue sections underwent gradient ethanol dehydration. Sections were incubated with 3 mL/L methanol–H_2_O_2_ for 15 min to block endogenous peroxidase activity. Antigen retrieval was achieved by incubating sections with 1 g/L trypsin at 37 °C for 30 min. Sections were blocked with 5% skim milk and incubated overnight with primary antibodies against: anti-cleaved caspase-1 (ab179515, Abcam) and GSDMD (ab209845, Abcam). Secondary antibody incubation with Immunoglobulin G (ab6721, Abcam) was conducted at 37 °C for 1 h. Development was carried out with 3,3'-Diaminobenzidine, followed by counterstaining with hematoxylin.

### Data analysis

Results are expressed as mean ± standard deviation (SD) from independent experiments. Cell experiments were conducted with at least three biological replicates and three technical repeats. Animal experiments were conducted with ten biological replicates. Statistical analysis was performed using Student’s *t*-test for comparisons between two groups, and one-way Analysis of Variance for comparisons among three or more groups, with Tukey’s Honestly Significant Difference test for post-hoc comparisons between groups. Statistical analysis was conducted using GraphPad Prism 9.0 (GraphPad Software, Inc., USA). Differences were considered statistically significant at *P < 0.05.

## Results

### Elevated expression of circSOD2 in NAFLD

**Fig. 1:**
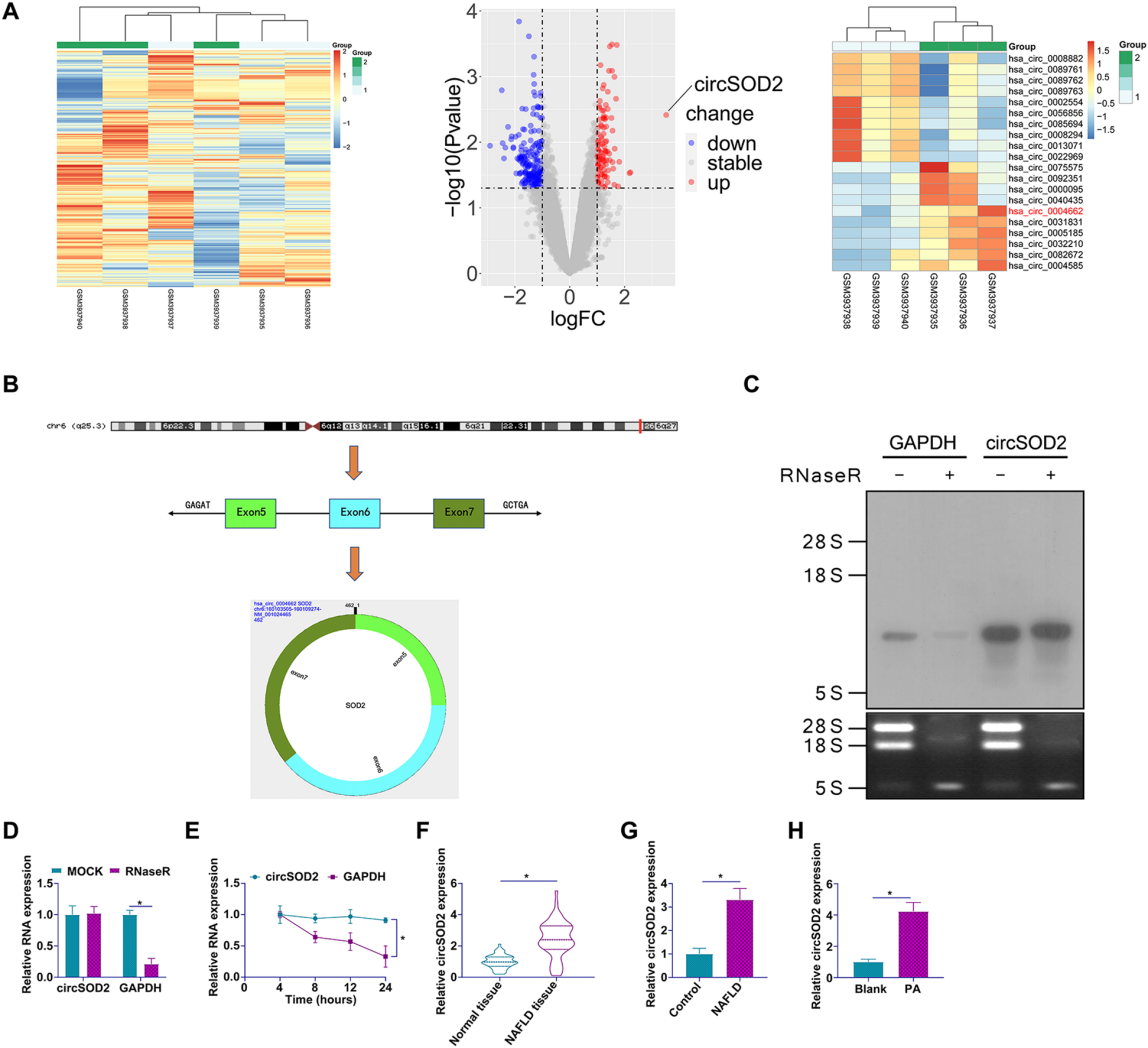
Elevated expression of circSOD2 in NAFLD. **A** Heatmaps depicting top ten highly and lowly expressed circRNAs from circMine transcriptome data; **B** Circular structure and gene information of circSOD2; **C** and **D** validation of the circular structure of circSOD2 via RNase R and Northern blot assays; **E** assessment of circSOD2 stability using actinomycin D assay; **F** quantification of circSOD2 expression in liver tissues of NAFLD patients by RT-qPCR; **G** analysis of circSOD2 expression in liver tissues of NAFLD rats by RT-qPCR; **H** expression of circSOD2 in PA-treated AML12 cells evaluated by RT-qPCR; data are presented as mean  ± SD (*n* = 3).*P < 0.05

To elucidate the aberrant expression of circRNAs in NAFLD, transcriptomic data from three normal liver tissue samples and three liver samples with NASH-related cirrhosis were harvested from the circMine database (http://www.biomedical-web.com), under the accession numbers GEO: GSM3937935-40. This analysis unveiled 107 circRNAs with significantly elevated expression and 181 circRNAs with markedly reduced expression (Additional file 4: Table S3). Figure 1A displays the top ten circRNAs with the highest and lowest expression levels. hsa_circ_0004662 emerged as the circRNA of interest due to its highest log2 fold change (log2 fold change = 3.5187). Further investigation on the circBase bioinformatics platform revealed that hsa_circ_0004662, with a spliced sequence length of 462 bp, is composed of exons 5–7 of the SOD2 gene. Consequently, hsa_circ_0004662 was designated as circSOD2. Subsequent validation of circSOD2’s circular structure was conducted. RNase R assays, Northern blot analysis, and actinomycin D experiments indicated that while RNase R and Actinomycin D treatments diminished GAPDH expression and stability, respectively, circSOD2 exhibited resistance to RNase R and Actinomycin D (Fig. 1C–E). Further examination of circSOD2 expression in NAFLD patients and in both in vivo and in vitro models demonstrated that circSOD2 was significantly upregulated in NAFLD compared to normal controls (Fig. 1F–H). Collectively, these findings suggest that circSOD2 is aberrantly overexpressed in NAFLD and may play a pivotal role in the progression of the disease.

### Targeted depletion of circSOD2 mitigates PA-induced pyroptotic cascades in hepatocytes

**Fig. 2:**
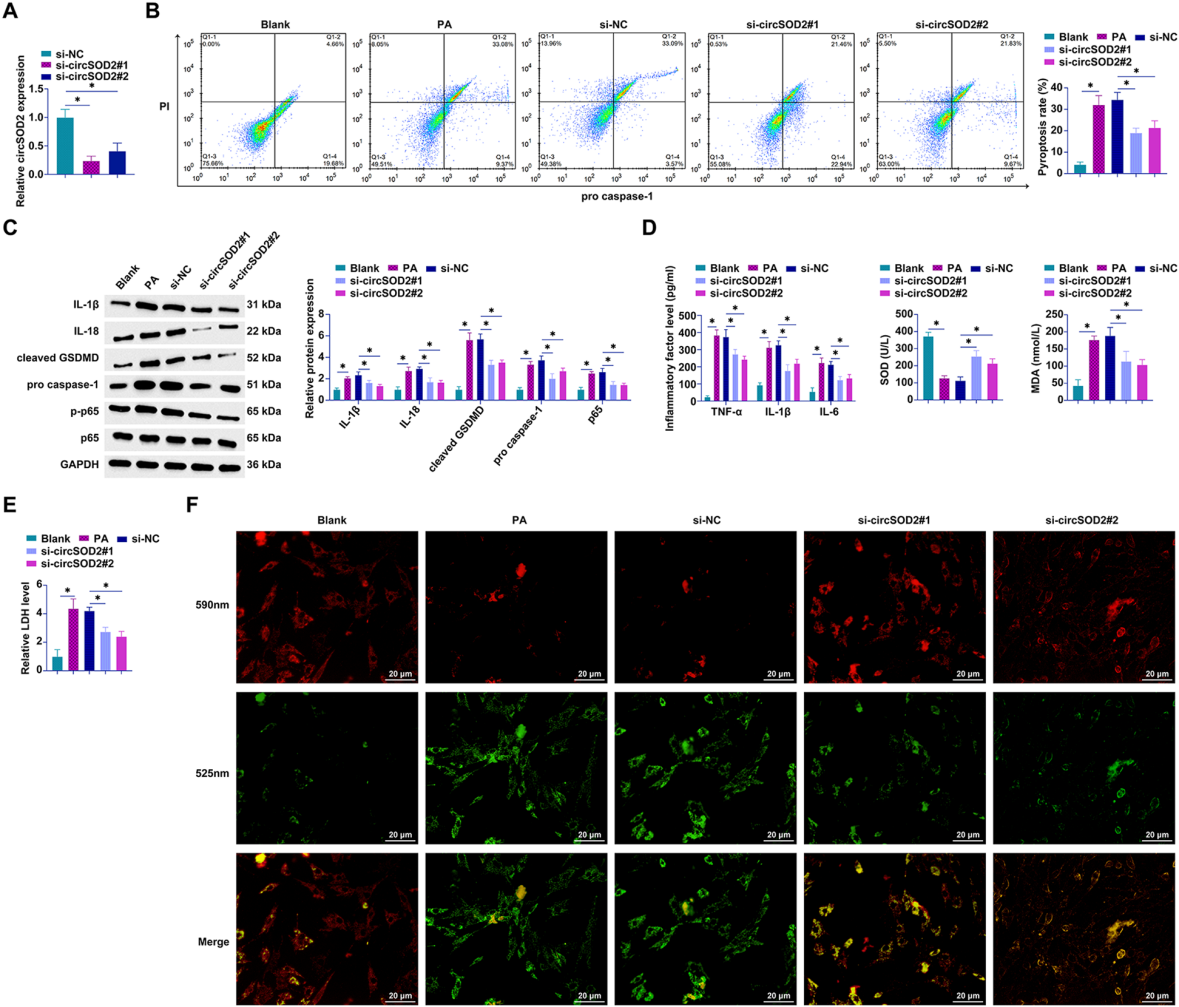
Knockdown of circSOD2 attenuates PA-induced hepatocyte pyroptosis. transfection of si-circSOD2#1 and si-circSOD2#2 into PA-treated AML12 cells. **A** Detection of circSOD2 expression by RT-qPCR; **B** pyroptotic cells assessed via flow cytometry; **C** expression of pyroptosis-related proteins IL-1β, IL-18, GSDMD, cleaved caspase-1, and phosphorylated p65 analyzed by Western blot; **D** levels of TNF-α, IL-1β, IL-6, SOD, and MDA determined by ELISA or conventional assay kits; **E** LDH release measured using assay kits; **F** MMP assessed by JC-1 staining (100 × , 20 μm); data are presented as mean  ± SD (*n* = 3).*P < 0.05

Accumulating evidence underscores the pivotal role of pyroptosis, a lytic form of programmed cell death characterized by its involvement in the immune response against intracellular pathogens, in exacerbating pathophysiological states such as chronic hepatitis and hepatic fibrosis [24]. Prompted by these insights, our investigation focused on delineating the regulatory capacity of circSOD2 in the pyroptosis pathway within the milieu of NAFLD. Utilizing a PA-induced in vitro model, we employed siRNA targeting circSOD2 to modulate its expression dynamically. Effective silencing of circSOD2 was achieved, as evidenced by the marked reduction in its levels post-transfection with si-circSOD2 constructs #1 and #2 (Fig. 2A). Initial assessments employing flow cytometric analysis revealed a significant augmentation in the proportion of hepatocytes undergoing pyroptosis following PA challenge, an effect conspicuously ameliorated by circSOD2 knockdown (Fig. 2B). This observation prompted further evaluation of molecular markers integral to the pyroptotic pathway. Western blot analyses delineated a significant upsurge in the expression of IL-1β, IL-18, GSDMD, cleaved caspase-1, and phosphorylated NF-kB p65 in response to PA, an upregulation effectively subdued by circSOD2 depletion (Fig. 2C). Furthermore, the quantification of inflammatory mediators and oxidative stress indices via ELISA and conventional assay kits revealed PA-induced elevations in TNF-α, IL-1β, IL-6, and MDA alongside a suppression of SOD activity, alterations significantly reversed upon circSOD2 knockdown (Fig. 2D). In tandem, evaluations of cellular LDH release and MMP fluctuations postulated PA’s propensity to facilitate LDH release and compromise MMP integrity, phenomena robustly mitigated by the targeted ablation of circSOD2 (Fig. 2E, F). Collectively, these findings illuminate the therapeutic potential of circSOD2 depletion in PA-induced hepatocellular pyroptosis.

### circSOD2 acts as a competitive miRNA sponge for miR-532-3p in cytoplasmic regulation

**Fig. 3:**
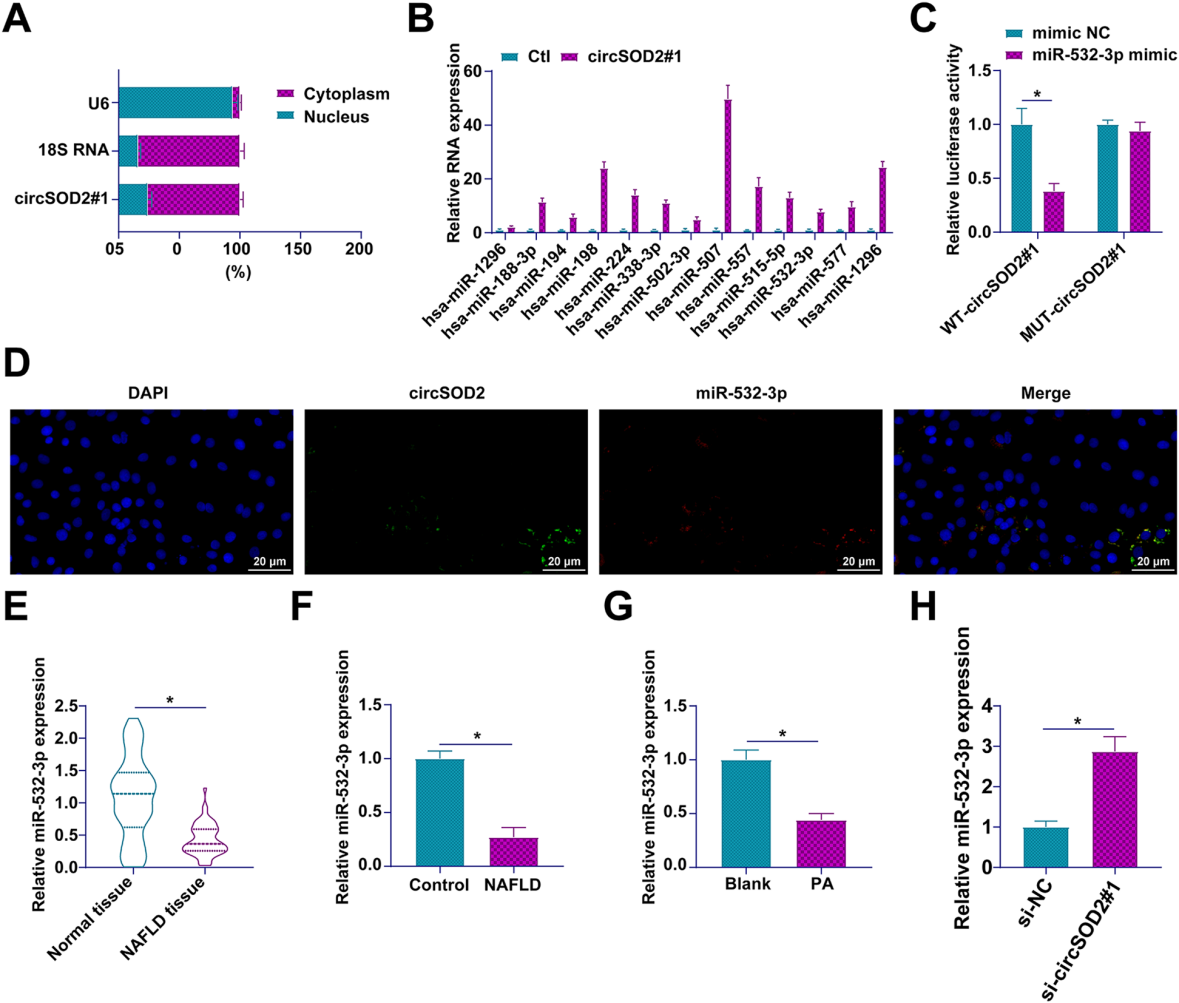
circSOD2 competitively binds to miR-532-3p. **A** subcellular localization of circSOD2; **B** identification of 13 potential miRNAs interacting with circSOD2 via circRIP and bioinformatics analysis; **C** targeted interaction between circSOD2 and miR-532-3p validated by dual-luciferase reporter assay; **D** co-localization of circSOD2 and miR-532-3p assessed by FISH; **E** expression of miR-532-3p in liver tissues of NAFLD patients analyzed by RT-qPCR; **F** miR-532-3p expression in liver tissues of NAFLD rats assessed by RT-qPCR; **G** evaluation of miR-532-3p expression in PA-treated AML12 cells by RT-qPCR; **H** impact of circSOD2 knockdown on miR-532-3p levels determined by RT-qPCR; Data are presented as mean ± SD (*n* = 3).*P < 0.05

In the cytoplasm, circRNAs frequently act as potent molecular sponges, sequestering miRNAs to modulate post-transcriptional gene regulation. Our investigation initially probed the subcellular localization of circSOD2 in AML12 hepatocytes, revealing a pronounced cytoplasmic presence indicative of its potential role in miRNA interaction (Fig. 3A). Pursuing this line of inquiry, circRIP assays were employed to isolate miRNAs associating with circSOD2, identifying thirteen candidates with predicted binding sites as per the circInteractome database (Fig. 3B). Subsequent screening through dual-luciferase reporter assays pinpointed a specific miRNA, miR-532-3p, that exhibits a definitive regulatory interaction with circSOD2. This was evidenced by a decrease in luciferase activity upon co-transfection of cells with wild-type circSOD2 and a miR-532-3p mimic, a phenomenon not observed with mutations in the circSOD2 binding site (Fig. 3C). FISH assays further confirmed the colocalization of circSOD2 and miR-532-3p within the cytoplasm of AML12 cells, solidifying the notion of their direct interaction (Fig. 3D). Extending our analysis to the expression dynamics of miR-532-3p within the milieu of NAFLD, we observed a consistent underexpression across clinical NAFLD samples, NAFLD rat models, and PA-induced AML12 hepatocyte cultures (Fig. 3E–G). Intriguingly, circSOD2 knockdown led to a significant elevation in miR-532-3p levels within AML12 cells (Fig. 3H), underscoring the sponge-like functionality of circSOD2 in sequestering miR-532-3p.

### miR-532-3p overexpression counteracts PA-induced pyroptotic pathways in hepatocytes

**Fig. 4:**
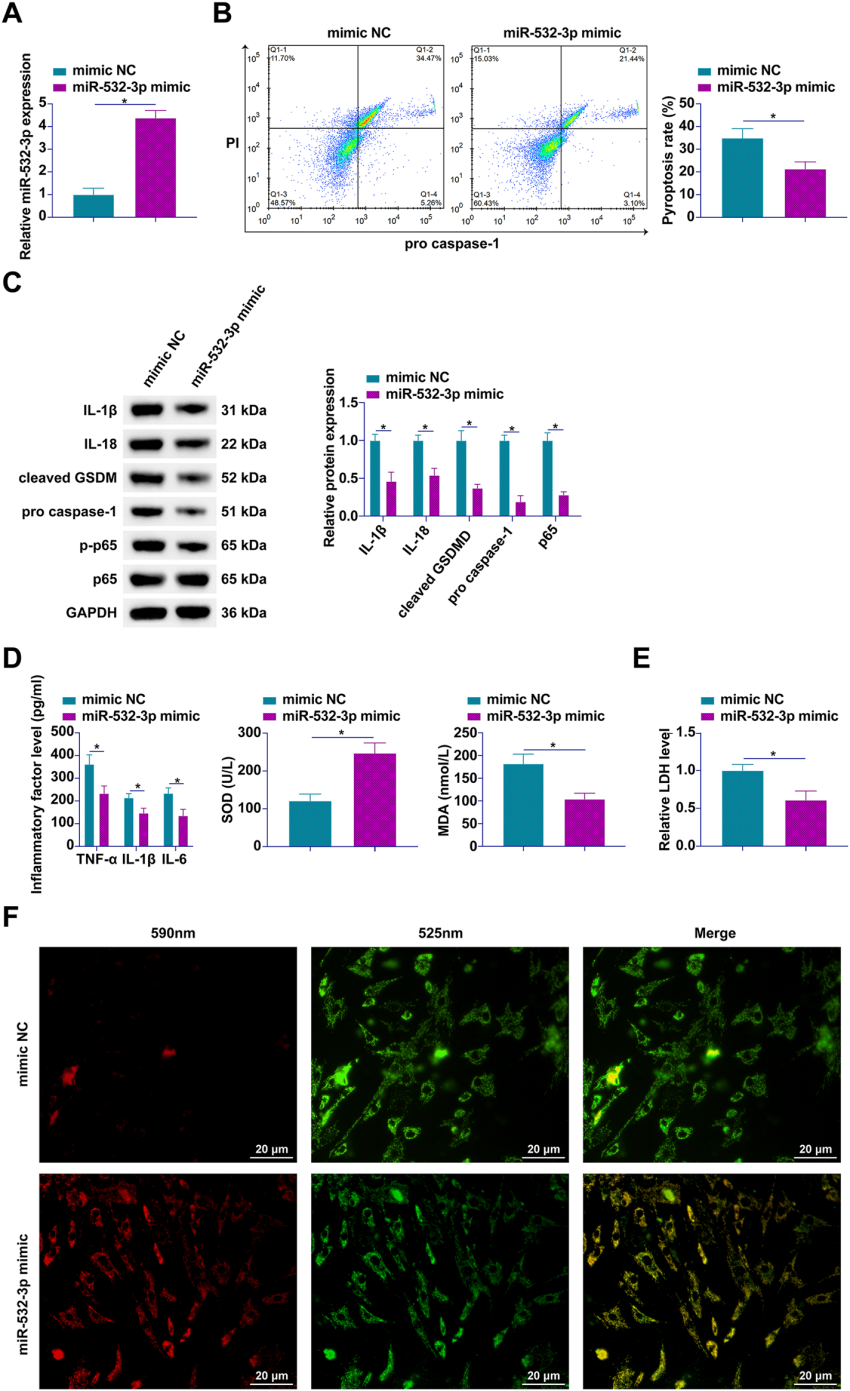
Overexpression of miR-532-3p alleviates PA-induced hepatocyte pyroptosis. Introduction of miR-532-3p mimic into PA-treated AML12 cells. **A** miR-532-3p expression monitored by RT-qPCR;**B** pyroptotic cells quantified by flow cytometry;** C** Western blot analysis of pyroptosis-associated proteins IL-1β, IL-18, pro IL-1β, pro IL-18, cleaved GSDMD, cleaved caspase-1, and phosphorylated p65;** D** concentrations of TNF-α, IL-1β, IL-6, SOD, and MDA measured by ELISA or standard assay kits;** E** LDH release evaluated using assay kits;** F** MMP assessed by JC-1 staining (100 × , 20 μm); data are represented as mean ± SD (*n* = 3). *P < 0.05

In an effort to elucidate the regulatory mechanisms of miR-532-3p in hepatocyte pyroptosis, our study introduced miR-532-3p mimics into AML12 cells subjected to PA insult. Figure 4A showcases the successful upregulation of miR-532-3p, achieved through the transfection of these mimics. Utilizing flow cytometry to assess the extent of cell pyroptosis, we observed that the overexpression of miR-532-3p markedly reduced the incidence of pyroptosis in hepatocytes exposed to PA (Fig. 4B). Further examination at the molecular level revealed an increase in the expression of key pyroptotic and inflammatory markers, including IL-1β, IL-18, cleaved caspase-1, GSDMD, and phosphorylated p65, in response to miR-532-3p overexpression (Fig. 4C). Complementary to these molecular insights, ELISA outcomes demonstrated that miR-532-3p overexpression notably enhanced the levels of SOD in the supernatant, while significantly decreasing the concentrations of TNF-α, IL-1β, IL-6, and MDA (Fig. 4D). Moreover, miR-532-3p overexpression was associated with a reduction in LDH release and an augmentation of MMP within AML12 cells (Fig. 4E, F). Collectively, these findings position miR-532-3p mimic as a pivotal modulator capable of alleviating PA-induced hepatocyte pyroptosis.

### circSOD2 depletion-driven attenuation of pyroptosis is counteracted by miR-532-3p inhibition in AML12 cells

**Fig. 5:**
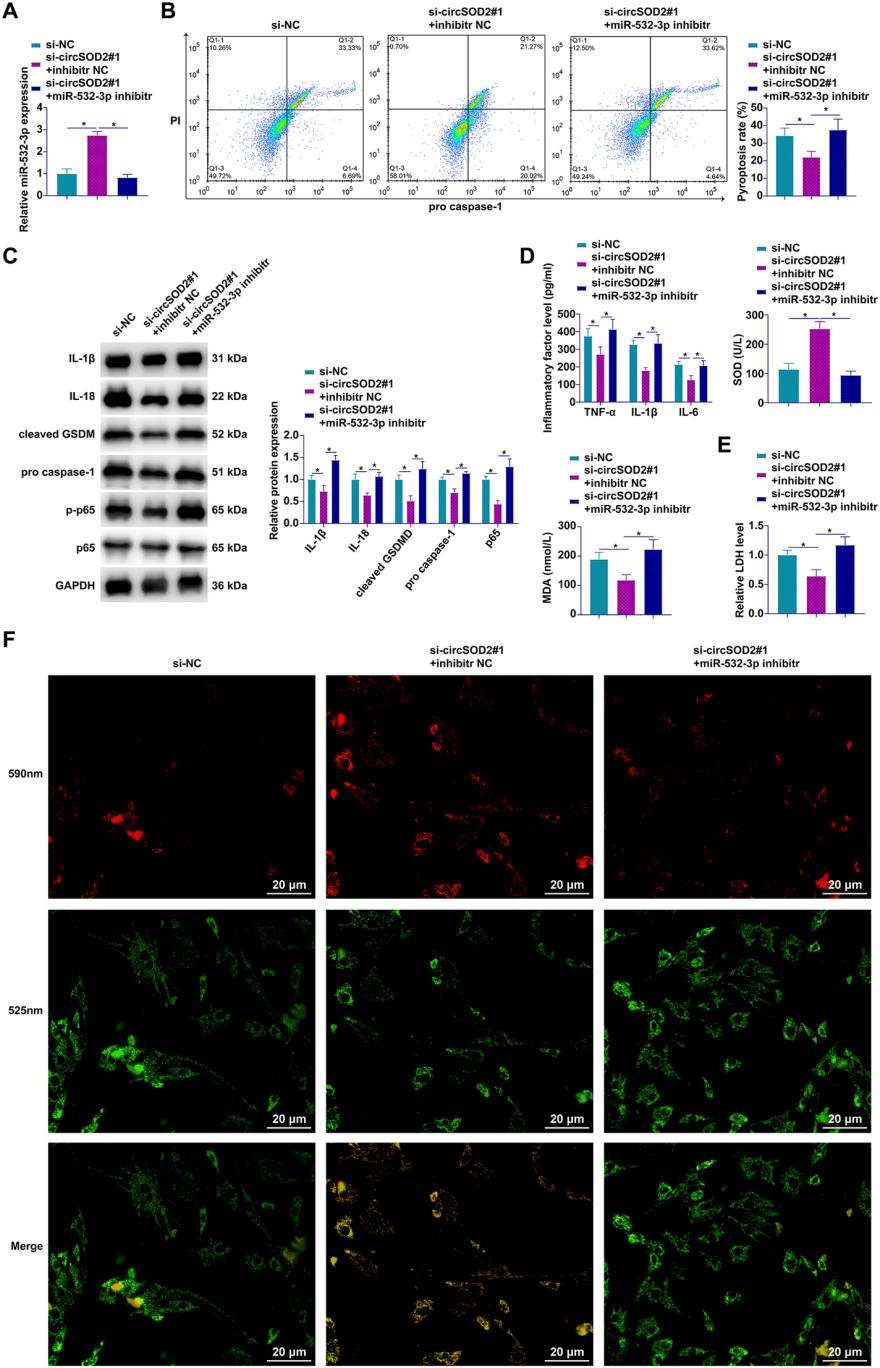
The inhibitory effect of circSOD2 knockdown on AML cell pyroptosis is reversed by knockdown of miR-532-3p. Transfection of miR-532-3p inhibitor into circSOD2-knockdown AML12 cells. **A** Measurement of miR-532-3p expression by RT-qPCR; **B** detection of pyroptotic cells by flow cytometry; **C** analysis of pyroptosis-related proteins IL-1β, IL-18, GSDMD, cleaved caspase-1, and phosphorylated p65 by Western blot; **D** levels of TNF-α, IL-1β, IL-6, SOD, and MDA determined by ELISA or conventional assay kits; **E** Assessment of LDH release with assay kits; **F** MMP evaluated by JC-1 staining (100 × , 20 μm); data are presented as mean  ± SD (*n* = 3) *P < 0.05.

Investigating the complex interplay between circSOD2 and miR-532-3p, our study delved into their roles in the modulation of pyroptosis within AML12 cells. To this end, circSOD2 was silenced, followed by the introduction of a miR-532-3p-specific inhibitor. The results, depicted in Fig. 5A, confirm a marked suppression of miR-532-3p post-inhibitor transfection. Utilizing flow cytometry and Western blot analysis, our research unveiled that the reduction in pyroptosis observed upon circSOD2 knockdown—characterized by a lowered rate of pyroptotic cells and a decrease in the expression of key pyroptosis markers—was effectively reversed by miR-532-3p inhibition (Fig. 5B, C). Biochemical assays further illustrated this dynamic interaction. circSOD2 silencing led to decreased levels of pro-inflammatory cytokines (TNF-α, IL-1β, IL-6) and oxidative stress marker MDA, concurrently increasing SOD activity. Intriguingly, miR-532-3p suppression negated these biochemical changes induced by circSOD2 knockdown, reinstating the original pro-pyroptotic environment (Fig. 5D, E). Additionally, JC-1 staining to assess MMP highlighted an increase in MMP levels upon circSOD2 depletion, a phenomenon reversed by the downregulation of miR-532-3p (Fig. 5F). These findings articulate a critical molecular dialogue between circSOD2 and miR-532-3p, delineating their significant influence on the regulation of pyroptosis in AML12 cells.

### miR-532-3p/TXNIP axis as a novel regulatory pathway in NAFLD

**Fig. 6:**
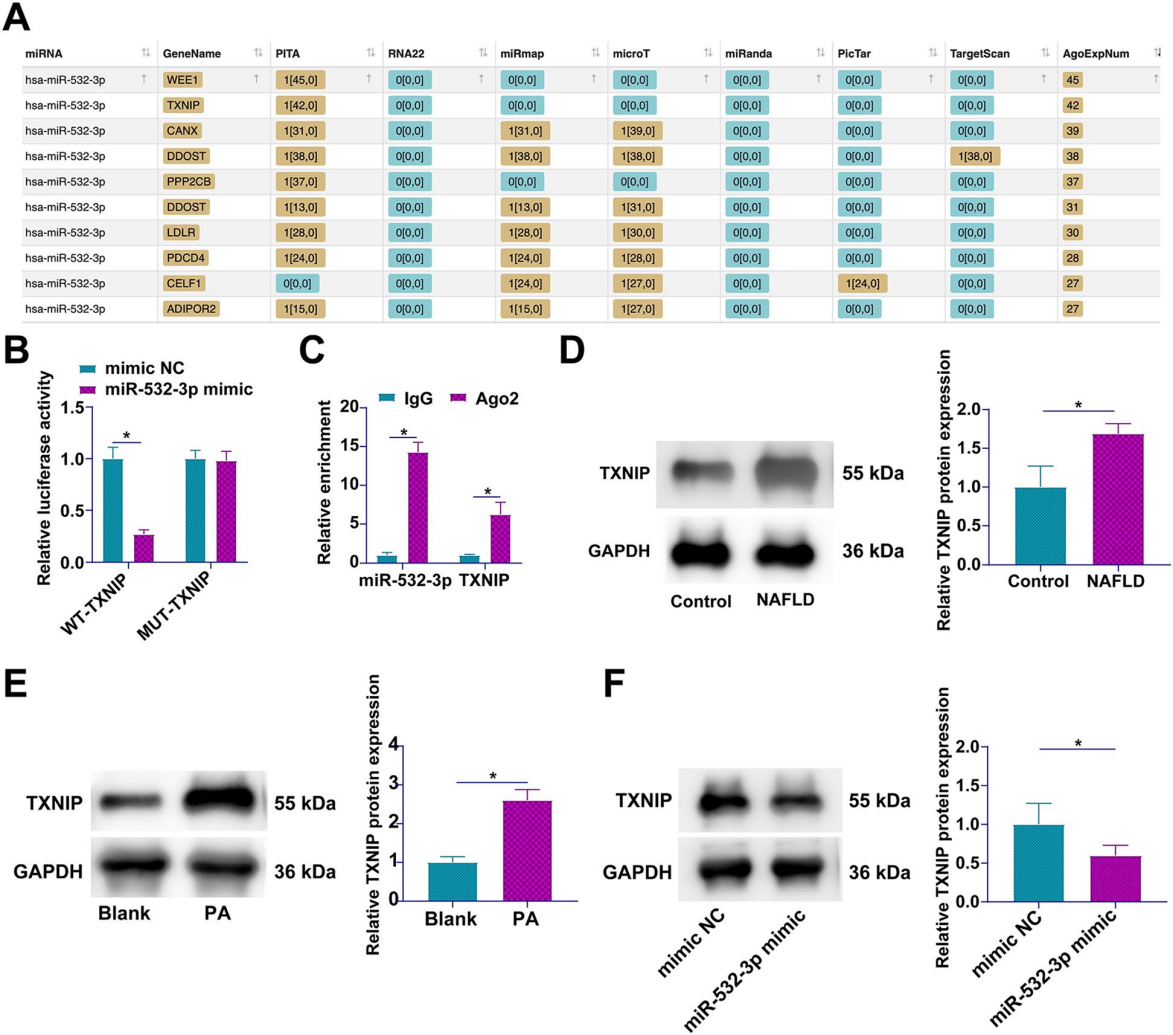
TXNIP is a downstream target gene of miR-532-3p. **A** Ten mRNAs with the highest AGO CLIP-seq sequencing data with miR-532-3p predicted by bioinformatics resource [https://starbase.sysu.edu.cn/]; **B** Targeted interaction between miR-532-3p and TXNIP validated by dual-luciferase reporter assay; **C** Binding relationship between miR-532-3p and TXNIP explored by RIP assay; **D** Expression of TXNIP in liver tissues of NAFLD patients analyzed by Western blot; **E** TXNIP expression in liver tissues of NAFLD rats assessed by Western blot; **F **effect of miR-532-3p overexpression on TXNIP levels determined by Western blot; data are represented as mean ± SD (*n* = 3). *P < 0.05

Harnessing the analytical prowess of bioinformatics, specifically the AGO CLIP-seq data repository available on https://starbase.sysu.edu.cn/, our study embarked on elucidating the intricate web of miR-532-3p interactions. Among the panoply of mRNA targets identified (Fig. 6A), TXNIP emerged as a focal point due to its critical role in orchestrating the activation of the NLRP3 inflammasome [25], a cornerstone in the inflammatory cascade implicated in myriad pathologies. This premise was further explored through the generation and application of dual-luciferase reporter constructs, informed by predictive modeling of miR-532-3p and TXNIP interaction sites (Fig. 6B). The experimental outcomes revealed a discernible suppression of luciferase activity following the co-transfection with WT-TXNIP and miR-532-3p mimics, an effect that was conspicuously absent in MUT-TXNIP, thereby affirming the specificity of the interaction (Fig. 6C). The specificity and functional relevance of the miR-532-3p/TXNIP interaction were further corroborated by RIP assays, demonstrating significant enrichment of TXNIP and miR-532-3p within Ago2 complexes, pivotal components of the RNA-induced silencing complex (Fig. 6D). Extending our inquiry to the pathophysiological context of NAFLD, TXNIP expression was systematically assessed. A consistent pattern of TXNIP upregulation was observed across clinical NAFLD specimens, in vivo, and in vitro models (Fig. 6E–G). Intriguingly, this upregulated expression profile was effectively attenuated upon the introduction of miR-532-3p mimics, indicating a direct regulatory impact of miR-532-3p on TXNIP expression levels (Fig. 6H). These results unveil the miR-532-3p/TXNIP axis as a heretofore underappreciated but pivotal regulatory pathway influencing the inflammatory landscape of NAFLD.

### TXNIP knockdown ameliorates PA-induced hepatocyte pyroptosis

**Fig. 7:**
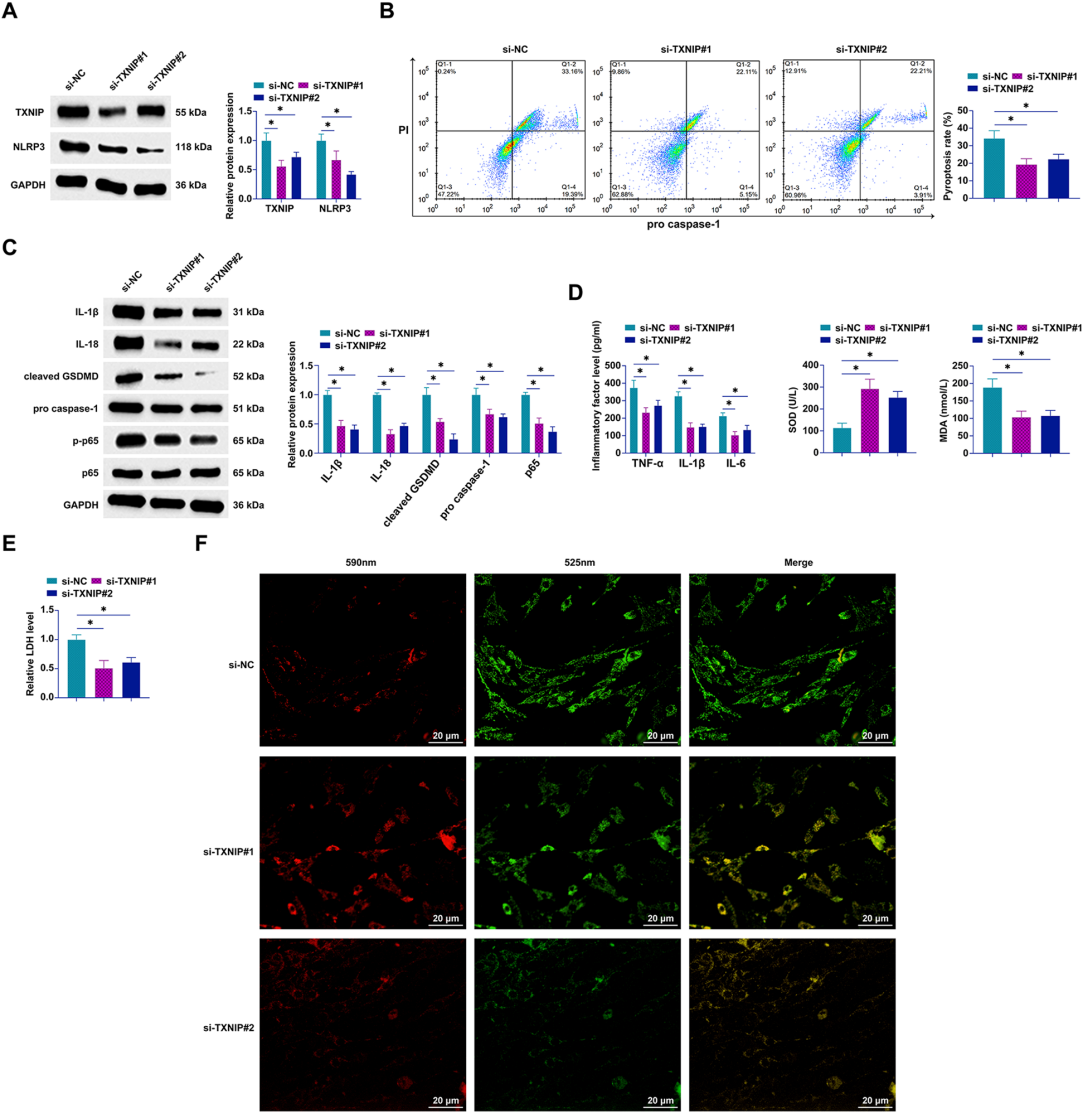
Knockdown of TXNIP prevents PA-induced hepatocyte pyroptosis by downregulating NLRP3. Transfection of si-TXNIP#1 and si-TXNIP#2 into PA-treated AML12 cells. **A** TXNIP and NLRP3 expression analyzed by Western blot; **B** pyroptotic cells quantified by flow cytometry; **C** expression of pyroptosis-associated proteins IL-1β, IL-18, GSDMD, cleaved caspase-1, and phosphorylated p65 assessed by Western blot; **D** concentrations of TNF-α, IL-1β, IL-6, SOD, and MDA measured by ELISA or standard assay kits; **E** LDH release evaluated using assay kits; **F** MMP assessed by JC-1 staining (100 × , 20 μm); data are presented as mean ± SD (*n* = 3). *P  < 0.05

To explore the functional role of TXNIP in hepatocyte pyroptosis, our investigation employed siRNA-mediated silencing of TXNIP (si-TXNIP#1 and si-TXNIP#2) in PA-treated AML12 cells. The efficacy of TXNIP knockdown in modulating the expression of both TXNIP and NLRP3 was confirmed, as depicted in Fig. 7A, indicating a successful suppression of these key pyroptosis regulators. Subsequent analyses through flow cytometry and Western blot techniques provided compelling evidence of the anti-pyroptotic effect of TXNIP silencing. Specifically, AML12 cells exhibited a marked reduction in pyroptosis rates post-TXNIP knockdown, accompanied by a pronounced decrease in the expression of pyroptosis-associated proteins including IL-1β, IL-18, GSDMD, cleaved caspase-1, and phosphorylated p65 (Fig. 7B, C). This phenotypic modulation underscores the critical role of TXNIP in the cellular pyroptosis machinery. Further biochemical assessments revealed that TXNIP depletion not only mitigated the levels of inflammatory cytokines (TNF-α, IL-1β, IL-6) but also attenuated oxidative stress within the AML12 cell milieu (Fig. 7D). Moreover, the silencing of TXNIP effectively inhibited LDH release and enhanced MMP, indicative of improved cellular viability and function (Fig. 7E, F). By inhibiting TXNIP, we demonstrate a viable strategy to mitigate PA-induced hepatocyte pyroptosis.

### circSOD2 modulates PA-induced hepatocyte pyroptosis via the miR-532-3p/TXNIP axis

**Fig. 8:**
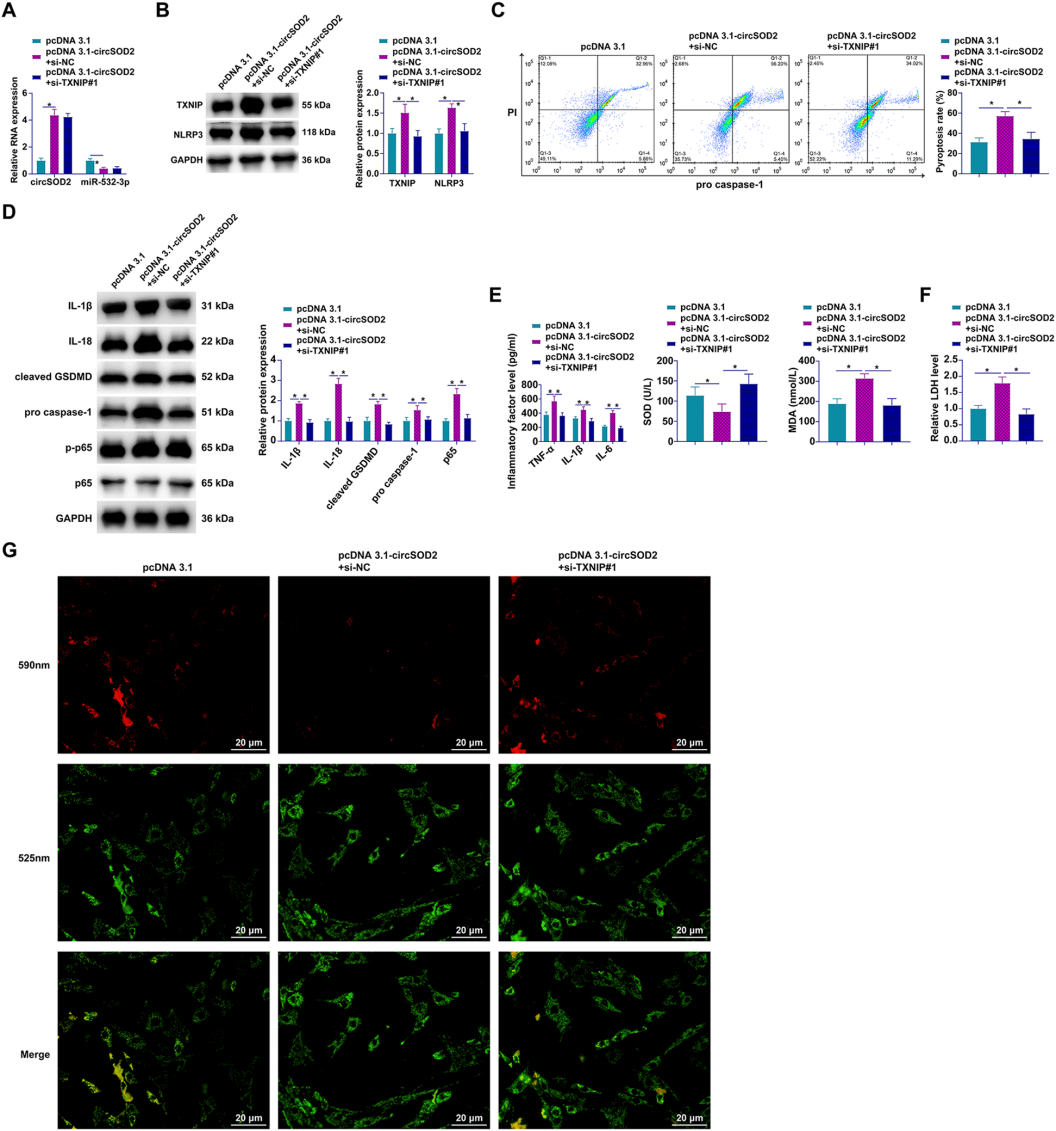
circSOD2 modulates PA-induced hepatocyte pyroptosis through the miR-532-3p/TXNIP axis. Co-transfection of pcDNA 3.1-circSOD2 and si-TXNIP#1 into PA-treated AML12 cells. **A** Expression of circSOD2 and miR-532-3p assessed by RT-qPCR; **B** TXNIP and NLRP3 expression evaluated by Western blot; **C** quantification of pyroptotic cells via flow cytometry; **D** analysis of pyroptosis-related proteins IL-1β, IL-18, GSDMD, cleaved caspase-1, and phosphorylated p65 by Western blot; **E** levels of TNF-α, IL-1β, IL-6, SOD, and MDA determined by ELISA or conventional assay kits; **F** LDH release measured using assay kits; **G** MMP assessed by JC-1 staining (100 × , 20 μm); data are represented as mean  ± SD (*n* = 3) *P < 0.05.

In further exploring the regulatory cascade, functional rescue experiments were conducted to assess the impact of the miR-532-3p/TXNIP axis on the biological functions of circSOD2. Co-transfection of pcDNA3.1-circSOD2 and si-TXNIP#1 into PA-stimulated AML12 cells illustrated that overexpression of circSOD2 upregulated TXNIP and NLRP3 expression while concurrently downregulating miR-532-3p, effects not observed with TXNIP silencing alone, which specifically decreased TXNIP and NLRP3 levels without altering circSOD2 and miR-532-3p expression (Fig. 8A, B). Flow cytometry and Western blot findings further corroborated that circSOD2 overexpression increased pyroptotic cell proportions and enhanced the expression of pyroptosis-related proteins (IL-1β, IL-18, GSDMD, cleaved caspase-1, p-p65), alterations that were effectively reversed by TXNIP knockdown (Fig. 8C, D). Moreover, circSOD2-induced upregulation of TNF-α, IL-1β, IL-6, MDA, and LDH levels, alongside a reduction in SOD activity, was mitigated by si-TXNIP, indicating that the deleterious effects of circSOD2 on cellular inflammatory and oxidative states are contingent upon TXNIP expression (Fig. 8E, F). JC-1 staining further confirmed that circSOD2 overexpression significantly diminished cellular MMP, an effect reversed by TXNIP silencing, thereby restoring mitochondrial integrity (Fig. 8G). Collectively, these findings delineate a novel regulatory mechanism by which circSOD2 influences PA-induced hepatocyte pyroptosis through the miR-532-3p/TXNIP axis.

### circSOD2 Attenuates NAFLD in Vivo via modulation of the miR-532-3p/TXNIP/NLRP3 signaling cascade

**Fig. 9:**
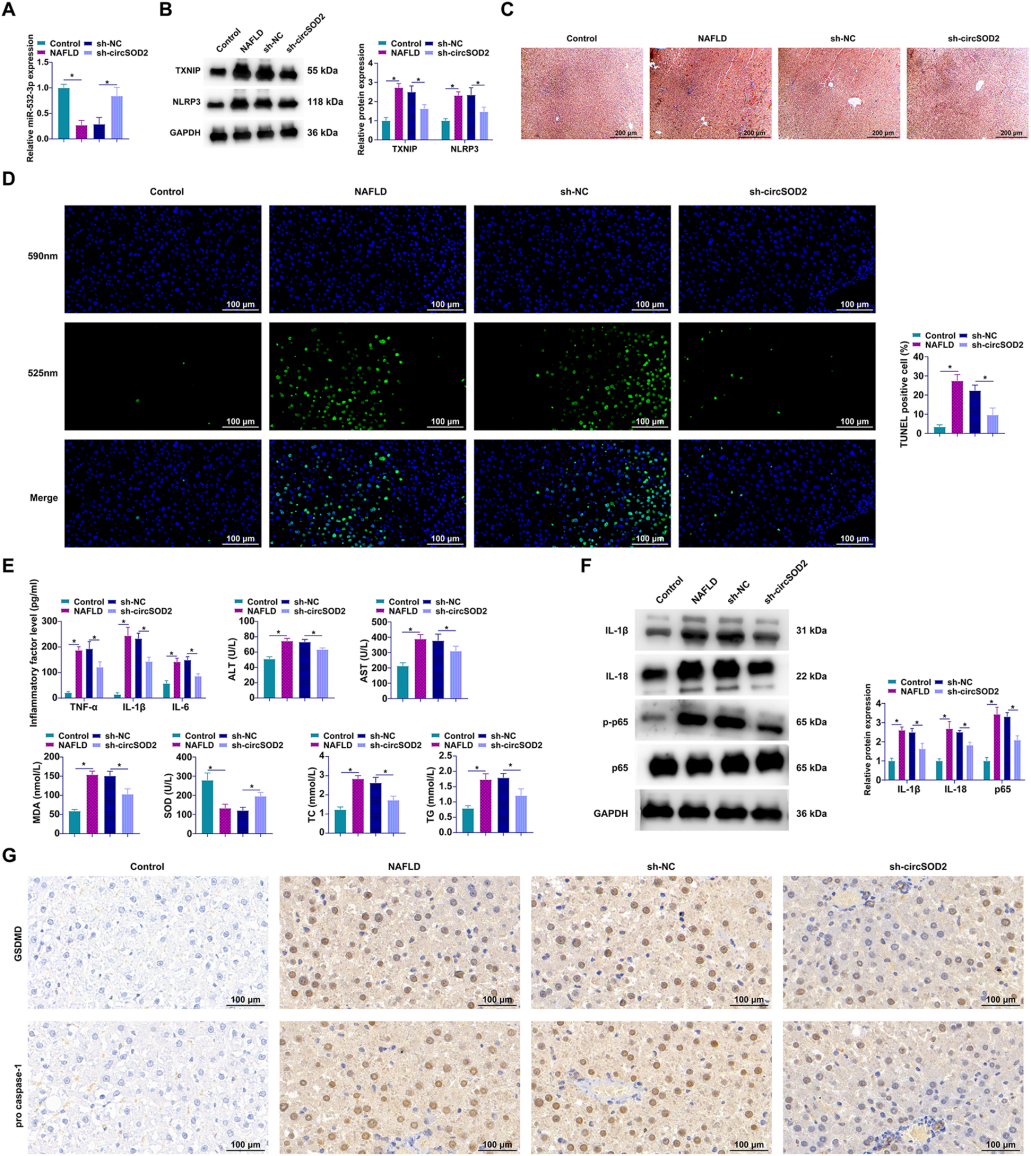
circSOD2 inhibits NAFLD in vivo. **A** miR-532-3p expression in rat liver tissues analyzed by RT-qPCR; **B** expression of TXNIP and NLRP3 in rat liver tissues assessed by Western blot; **C** representative images of Oil Red O staining of rat liver tissues (40 × , 200 μm); **D** TUNEL staining for apoptosis in rat liver tissues (40 × ,100 μm);** E** levels of serum ALT, AST, TC, TG, and liver TNF-α, IL-1β, IL-6, MDA, and SOD in rats; **F** Western blot analysis of IL-1β, IL-18, and phosphorylated p65 protein expression in rat liver tissues; **G** IHC detection of GSDMD and cleaved caspase-1 positivity rates in rat liver tissues; Data are presented as mean  ± SD (*n* = 10) *P < 0.05.

Extending our investigation to an in vivo context, we scrutinized the regulatory impact of circSOD2 on the pathophysiology of NAFLD. Employing RT-qPCR and Western blot analysis, we observed a pivotal modulation within the liver tissues of NAFLD rats: knockdown of circSOD2 significantly elevated miR-532-3p expression, concurrently with a pronounced suppression of TXNIP and NLRP3 levels (Fig. 9A, B). Histological assessment, facilitated by Oil Red O staining, vividly demonstrated the efficacy of circSOD2 silencing in mitigating hepatic lipid accumulation and steatosis, hallmark features of NAFLD (Fig. 9C). Furthermore, the application of TUNEL assay revealed a substantial reduction in hepatocyte apoptosis rates in circSOD2-depleted NAFLD rats (Fig. 9D). Biochemical analyses underscored the therapeutic potential of circSOD2 knockdown, evidencing decreased serum and hepatic concentrations of ALT, AST, TC, TG, TNF-α, IL-1β, IL-6, and MDA, alongside an enhancement in SOD activity (Fig. 9E). Complementary Western blot and IHC analyses furnished further insight into the anti-inflammatory and anti-pyroptotic effects of circSOD2 knockdown, revealing a decrease in the hepatic expression of IL-1β, IL-18, GSDMD, p-p65, and cleaved caspase-1 in circSOD2-silenced NAFLD models (Fig. 9F, G). Together, these findings illuminate the circSOD2-mediated orchestration of the miR-532-3p/TXNIP/NLRP3 axis as a potent modulator of NAFLD.

## Discussion

The prevalence of NAFLD continues to rise, posing significant health challenges both at individual and societal levels due to its increasing incidence [26]. Over recent years, non-coding RNAs have been demonstrated to play a crucial role in the progression of NAFLD [27]. By understanding the molecular mechanisms underlying the development and progression of NAFLD, novel therapeutic strategies can be devised to treat and prevent the condition. In this study, we focused on the molecular mechanisms of NAFLD-associated pyroptosis. Our results confirmed that circSOD2 competitively adsorbs miR-532-3p to mediate TXNIP expression, thereby regulating the NLRP3 inflammasome and activating downstream cascades to promote pyroptosis.

A core pathological feature of NAFLD is hepatic fat deposition. Recent works have confirmed the involvement of circRNAs in regulating disorders of hepatic lipid metabolism. For instance, circRNA-PI4KB facilitates hepatic lipid deposition in NAFLD by exporting miR-122 to extrahepatic cells [28]. Additionally, circRNA_0046366 suppresses hepatic steatosis through normalization of the PPAR signaling pathway [29]. Our findings further support the notion that circRNAs regulate lipid deposition. We observed that knockdown of circSOD2 effectively reduced the number of lipid droplets in NAFLD-afflicted rat livers. Notably, excessive accumulation of lipids in the liver induces a spectrum of pathologies, including oxidative stress, endoplasmic reticulum stress, and inflammatory responses [30, 31]. In this study, we demonstrated that knockdown of circSDO2 reduces hepatic MDA accumulation and inhibits phosphorylation of p65. Remarkably, this study is the first to unveil the role of circRNAs in NAFLD-associated pyroptosis. Pyroptosis is an inflammatory form of programmed cell death, primarily mediated by inflammasomes that activate a range of caspases, including Caspase-1, leading to cleavage and polymerization of various Gasdermin family members like GSDMD, causing cell perforation and subsequent death [32, 33]. Unlike apoptosis, pyroptosis occurs more rapidly and is accompanied by the release of pro-inflammatory cytokines (e.g., IL-1β, IL-18). The pyroptosis pathway is further activated following lipid deposition-induced oxidative stress, endoplasmic reticulum stress, and inflammatory responses. Activation of pyroptosis increases cell membrane permeability, leading to leakage of cellular contents, further activating immune cells, and promoting inflammatory responses, exacerbating liver damage [24, 34]. We found that knockdown of circSOD2 effectively blocked the pyroptosis process in hepatocytes induced by PA. Knockdown of circSOD2 reduced the expression of IL-1β, IL-18, GSDMD, and cleaved caspase-1, which could help maintain cell membrane permeability, thereby reducing the leakage of these inflammatory factors and preventing the activation of the inflammatory signal p65. We speculate that the inhibitory effect of circSOD2 on hepatocyte pyroptosis will ameliorate the hepatic pathological environment, potentially restoring the liver’s lipid metabolic capacity and improving hepatic lipid deposition.

Cytoplasmic circRNAs often function as competitive endogenous RNAs to bind miRNAs competitively, thus influencing gene regulation [35]. We discovered that circSOD2 is primarily expressed in the cytoplasm and binds to miR-532-3p to promote TXNIP expression. Functional rescue experiments further confirmed the significant role of the circSOD2/miR-532-3p/TXNIP axis in the pathogenesis of NAFLD. miRNAs are key regulators of liver physiological functions, including liver regeneration, lipid metabolism, apoptosis, and tissue development [36]. A multitude of dysregulated miRNAs, such as miR-21 and miR-140 [37, 38], have been reported in NAFLD. However, the role of miR-532-3p in NAFLD remains unclear. Current studies suggest that miR-532-3p plays a role in various inflammatory responses. For example, miR-532-3p targets BAK1 to suppress inflammation and apoptosis signals in human sarcopenia [39]. In this study, we also observed the anti-inflammatory effects of miR-532-3p. Upregulation of miR-532-3p effectively prevented phosphorylation of p65 and inhibited the expression of inflammatory factors. Additionally, our results indicated that the anti-inflammatory effects of miR-532-3p (Table 1) were significantly related to the pyroptosis pathway. Notably, miR-532-3p could also prevent pro-inflammatory properties of macrophages by reducing ASK1 levels and downstream phosphorylation of p38 MAPK [40]. Therefore, the inhibitory effect of circSOD2 on the biological functions of miR-532-3p could enhance hepatic macrophage M1 polarization, thus promoting inflammatory cascades.

**Table 1:** Primers

Genes	Primers
circSOD2	F: 5'-TGGGGATTGATGTGTGGGAG-3'
	R: 5'-CGTTAGGGCTGAGGTTTGTC-3'
miR-532-3p	F: 5'-CCTCCCACACCCAAGG-3'
	R: 5'- TGGTGTCGTGGAGTCG-3'
GAPDH	F: 5'-CATCAACGGGAAGCCCATC-3'
	R: 5'-CTCGTGGTTCACACCCATC-3'

*F* forward, *R* reverse, circSOD2, circular RNA SOD2; miR-532-3p, microRNA-532-3p; GAPDH, glyceraldehyde-3phosphate dehydrogenase

In this study, we identified the significant role of the circSOD2/miR-532-3p axis in hepatocyte pyroptosis as highly related to the TXNIP/NLRP3 axis. TXNIP exists intracellularly as an antioxidative protein [41]; upon oxidative stress or hyperglycemic conditions, TXNIP dissociates from its original antioxidative partner, Thioredoxin, and binds to the NLRP3 inflammasome [42]. This binding triggers the activation of the NLRP3 inflammasome, leading to the maturation and release of pro-inflammatory cytokines IL-1β and IL-18, sparking an inflammatory response [43–45]. CircSOD2 competitively adsorbed miR-532-3p, preventing the binding of miR-532-3p to TXNIP. This action upregulated TXNIP transcriptional activity, thereby enhancing downstream inflammatory cascades. Thus, inhibiting circSOD2 is an essential measure to control hepatic cell pyroptosis.

This study has limitations. Although we employed HFD-induced rat model and PA-treated hepatocyte model, which are effective in simulating certain aspects of NAFLD, they may not fully replicate the complex pathophysiology of human NAFLD. The development of human NAFLD is influenced by a myriad of genetic, metabolic, and environmental factors, which may not be fully mimicked in the current models. Future research should consider employing models closer to human pathophysiology, such as genetically edited animal models or liver cells from NAFLD patients, to understand the role of circRNAs more comprehensively in NAFLD. While this study successfully identified and investigated the role of circSOD2 in NAFLD, there are thousands of circRNAs in the human genome that may be involved in the development and progression of NAFLD in complex ways. Therefore, studying a single circRNA may not fully reveal the overall role of circRNAs in NAFLD. Future research should expand the exploration of other circRNAs’ roles in NAFLD and how they interact with miRNAs and other molecules to influence disease progression.

In summary, this study provided novel insights into the pathogenesis of NAFLD, demonstrating that circSOD2 competitively adsorbs miR-532-3p to activate the TXNIP/NLRP3 inflammatory signaling pathway, thereby promoting pyroptosis in NAFLD cells. Our research elucidates the critical role of the circSOD2/miR-532-3p/TXNIP/NLRP3 pathway in NAFLD, establishing a new molecular mechanism for the development and progression of NAFLD and offering alternative therapeutic avenues. Furthermore, validating the efficacy and safety of these molecular targets in human NAFLD patients through extensive clinical studies is essential.

### Supplementary Information

**Additional file 1: Fig S1.** Detection of cell transfection efficiency by flow cytometry.

**Additional file 2.** The sequences of siRNAs and the map of the pcDNA3.1 vector.

**Additional file 3: Table S2.** Patient clinical information.

**Additional file 4: Table S3.** Transcriptomic data from three normal liver tissue samples and three liver samples with NASH-related cirrhosis.

## Data Availability

Data is available from the corresponding author on request.
