# Supplementary figures and images for: Competitive adsorption of microRNA-532-3p by circular RNA SOD2 activates Thioredoxin Interacting Protein/NLR family pyrin domain containing 3 pathway and promotes pyroptosis of non-alcoholic fatty hepatocytes
Source: Eur J Med Res. 2024 Apr 24;29:250. doi: 10.1186/s40001-024-01817-4 (PMC11044449; doi:10.1186/s40001-024-01817-4)

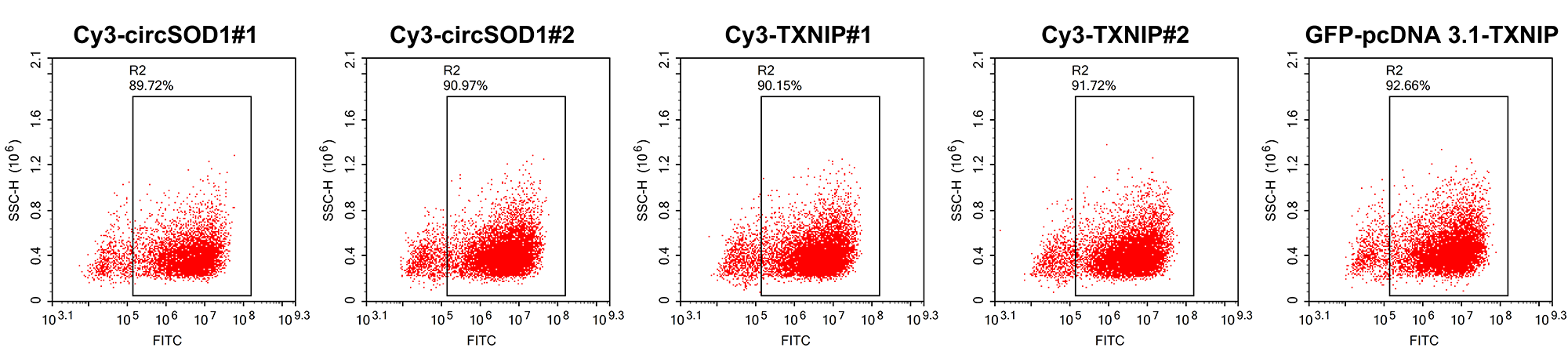

Supplement: Supplementary file 1 — Additional file 1: Fig S1. Detection of cell transfection efficiency by flow cytometry. [file 40001_2024_1817_MOESM1_ESM.tif]
