# Supplementary material for: Competitive adsorption of microRNA-532-3p by circular RNA SOD2 activates Thioredoxin Interacting Protein/NLR family pyrin domain containing 3 pathway and promotes pyroptosis of non-alcoholic fatty hepatocytes
Source: Eur J Med Res. 2024 Apr 24;29:250. doi: 10.1186/s40001-024-01817-4 (PMC11044449; doi:10.1186/s40001-024-01817-4)
Supplement: Supplementary file 2 — Additional file 2. The sequences of siRNAs and the map of the pcDNA3.1 vector. [file 40001_2024_1817_MOESM2_ESM.docx]

**Additional file**

**Table S1** siRNA sequence

| SiRNA | Sequence |
| --- | --- |
| circSOD2#1 | 5'-GTCATATCAATCATAGCATTTTC-3' |
| circSOD2#2 | 5'-TGGTTCCTTTGACAAGTTTAAGG-3' |
| TXNIP#1 | 5'-GGGAAAGAAGGCTTTTTCTCTGA-3' |
| TXNIP#1 | 5'-GGCTTTTTCTCTGAATTAGCTTA-3' |

**Plasmid map information**


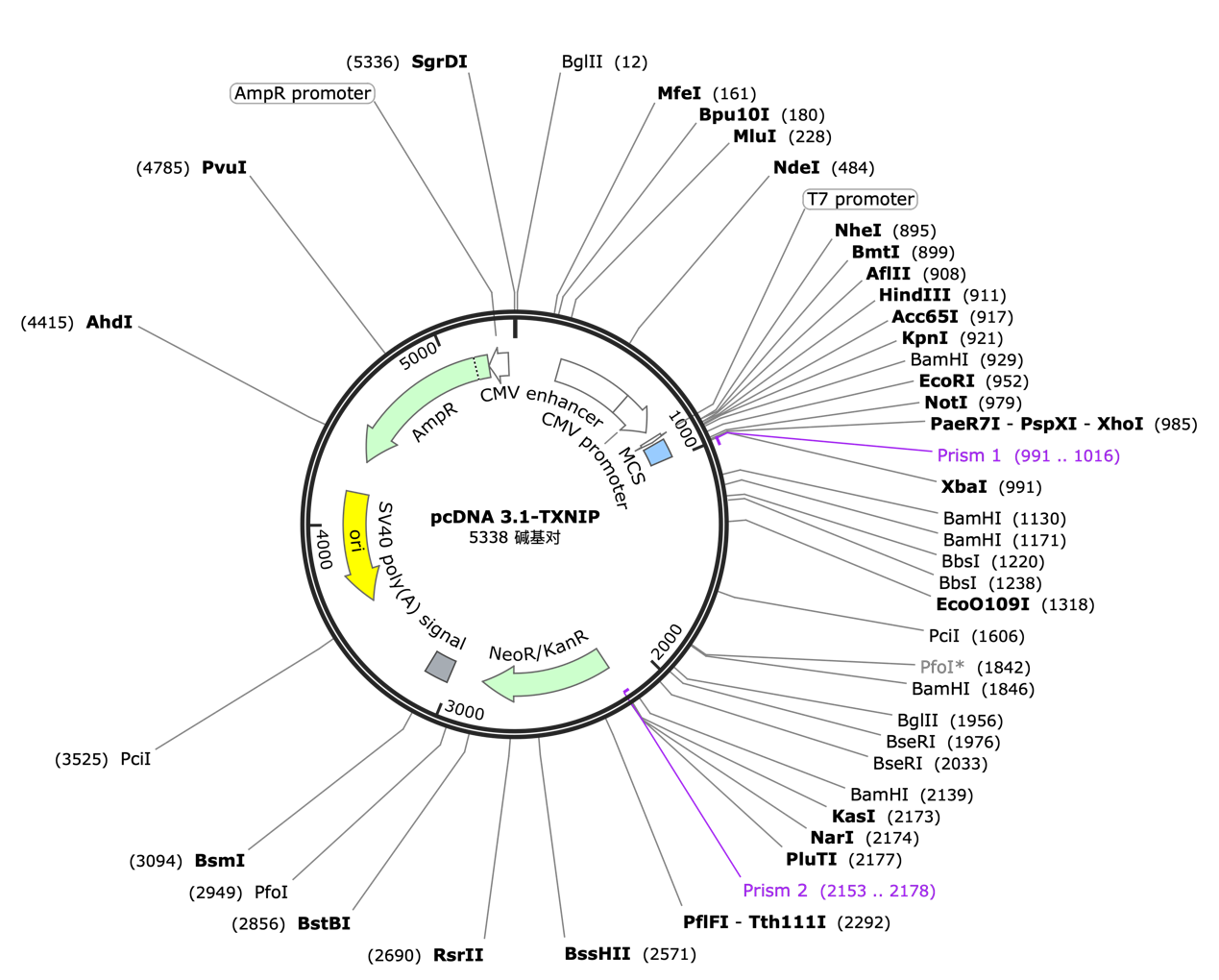


pcDNA 3.1-TXNIP plasmid map


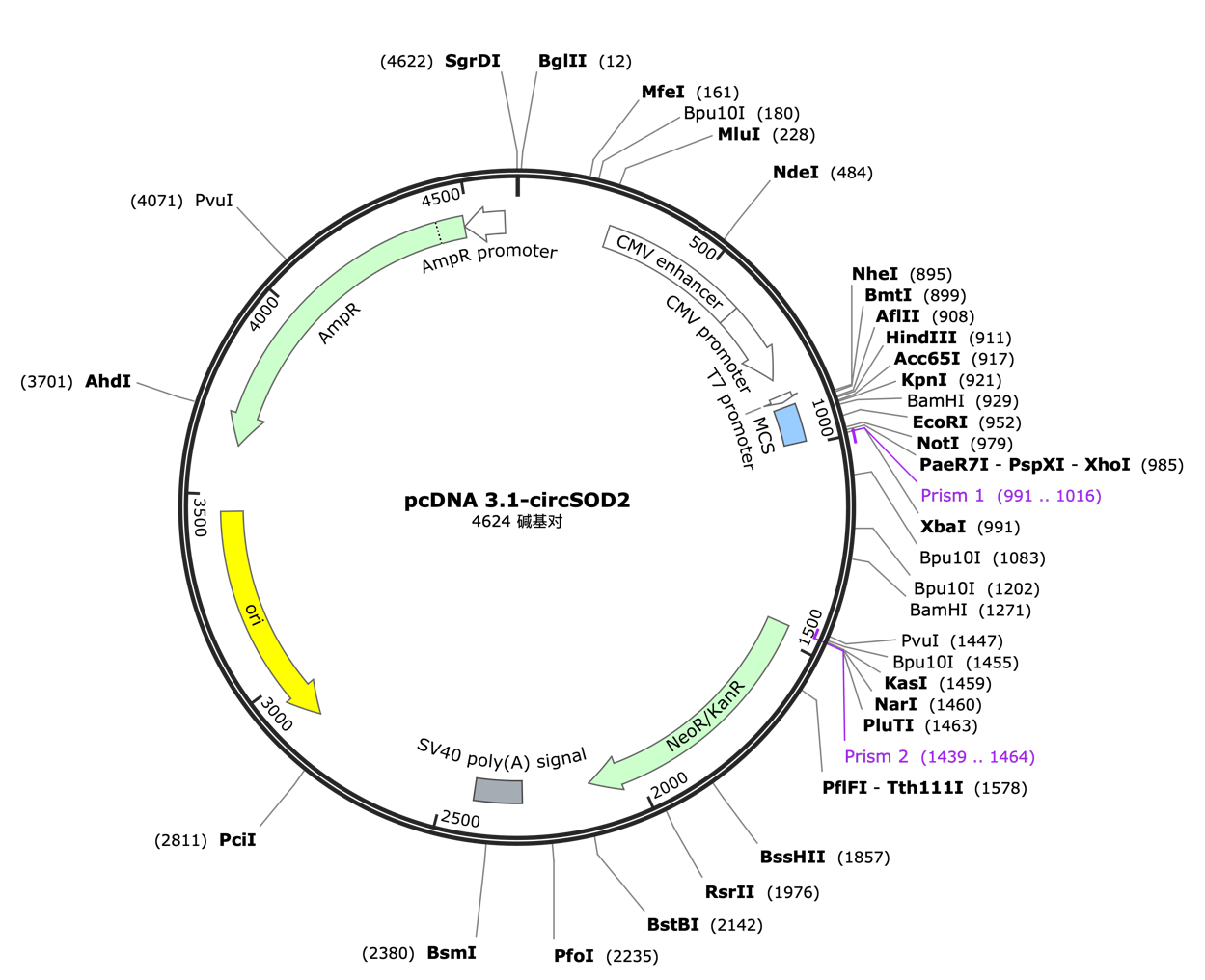


pcDNA 3.1-circSOD2 plasmid map
